# Supplementary material for: Epidemiological Trends of Haematological Malignancies in Belgium 2004–2018: Older Patients Show the Greatest Improvement in Survival
Source: Cancers (Basel). 2023 Sep 1;15(17):4388. doi: 10.3390/cancers15174388 (PMC10486374; doi:10.3390/cancers15174388)
Supplement: Supplementary file 1 [file cancers-15-04388-s001.zip › cancers-2541352-supplementary.pdf]

cancers MDPI

**Supplementary Materials:**

**Table S1**: Selection of ICD-O-3 codes for the 24 main HM types with respective abbreviations.

| Abbreviation | HM subtype | Topography | Histology | Behaviour | Differentiation |
|---|---|---|---|---|---|
| **HL** | Hodgkin lymphoma | Any | 9650-9655,9659,9661-9665,9667 | 3 | Any |
| **CLL/SLL** | B-cell chronic lymphocytic leukaemia / small lymphocytic lymphoma | Any | 9670,9823 | 3 | Any |
| **HCL** | Hairy cell leukaemia | Any | 9940 | 3 | Any |
| **Other MBCL** | Other Mature B-cell leukaemia and related lymphoma | Any | 9833 | 3 | Any |
| | | C420-C421 | 9591 | 3 | 6 |
| **IPD** | Immunoproliferative disease | Any | 9671,9760-9762,9764 | 3 | Any |
| **PCN** | Plasma cell neoplasm | Any | 9731-9734 | 3 | Any |
| **MZL** | Marginal zone lymphoma | Any | 9689,9699 | 3 | Any |
| **FL** | Follicular lymphoma and related lymphoma | Any | 9597,9690,9691,9695,9698 | 3 | Any |
| **MCL** | Mantle cell lymphoma | Any | 9673 | 3 | Any |
| **DLBCL** | Diffuse large B-cell lymphoma and related large B-cell lymphoma | Any | 9675,9678-9680,9684,9688,9712, 9735,9737,9738,9766 | 3 | Any |
| **BL** | Burkitt lymphoma / leukaemia | Any | 9687,9826 | 3 | Any |
| **pCTCL** | Primary cutaneous T-cell lymphoma | Any | 9700-9701,9709,9718,9726 | 3 | Any |
| **PNK/TCL** | Peripheral NK/T-cell lymphoma | Any | 9702,9705,9708,9714-9717,9719,9724,9827,9831,9834,9948 | 3 | Any |
| **ALL/LL** | Precursor lymphoid neoplasm or acute lymphoblastic leukaemia / lymphoma | Any | 9811-9819,9727-9729,9835-9837 | 3 | Any |
| **AML** | Acute myeloid leukaemia and related precursor neoplasm (includes acute leukaemia of ambiguous lineage) | Any | 9801,9805-9809,9840,9861,9865-9867, 9869-9874,9877-9879,9891,9895-9898, 9910-9912,9920,9930,9931,9984,9987 | 3 | Any |
| **CML** | Chronic myeloid leukaemia | Any | 9863,9875 | 3 | Any |
| **PV** | Polycythaemia vera | Any | 9950 | 3 | Any |
| **ET** | Essential thrombocythaemia | Any | 9962 | 3 | Any |
| **PMF** | Primary myelofibrosis | Any | 9961 | 3 | Any |
| **Other MPN** | Other MPN and related neoplasm | Any | 9960,9963-9968 | 3 | Any |
| **MCN** | Mast cell neoplasm | Any | 9740-9742,9749 | 3 | Any |
| | | Any | 9740,9741 | 1 | Any |
| **MDS** | Myelodysplastic syndrome | Any | 9980,9982,9983,9985,9986,9989, 9991-9993 | 3 | Any |
| **MDS/MPN** | Myelodysplastic / myeloproliferative neoplasm | Any | 9876,9945,9946,9975 | 3 | Any |
| **HDCN** | Histiocytic and dendritic cell neoplasm | Any | 9750,9751,9754-9759 | 3 | Any |
| | | Any | 9751-9753 | 1 | Any |
| **Not included** | Other leukaemia | Any | 9800,9860 | 3 | Any |
| | Other lymphoid neoplasm | Any | 9590,9596,9820,9832 | 3 | Any |
| | | C000-C419, C422-C809 | 9591 | 3 | 1-5 |
| | | C420-C421 | 9591 | 3 | 7-9 |

**Table S2**: Conditional 5-year relative survival by HM type, Belgium 2009-2018

| | Years after diagnosis | | | | | |
|---|---|---|---|---|---|---|
| **HM type** | **0** | **1** | **2** | **3** | **4** | **5** |
| **HL** | 88.4 | 94.2 | 95.6 | 96.5 | 96.6 | 96.7 |
| **CLL/SLL** | 90.4 | 92.3 | 91.5 | 91.2 | 90.6 | 89.2 |
| **HCL** | 96.7 | 98.1 | 99.4 | 98.9 | 99.4 | 102.0 |
| **Other MBCL** | 77.7 | 92.2 | 94.3 | 95.3 | 93.0 | 103.2 |
| **IPD** | 83.2 | 87.6 | 86.2 | 86.6 | 84.4 | 80.4 |
| **PCN** | 56.7 | 62.4 | 63.6 | 64.5 | 65.2 | 67.6 |
| **MZL** | 88.0 | 91.3 | 93.1 | 93.4 | 92.4 | 92.2 |
| **FL** | 90.5 | 91.9 | 92.9 | 93.3 | 93.9 | 94.7 |
| **MCL** | 62.6 | 68.2 | 69.1 | 70.4 | 71.0 | 73.0 |
| **DLBCL** | 61.1 | 82.9 | 89.5 | 92.1 | 91.5 | 90.9 |
| **BL** | 67.1 | 90.8 | 97.2 | 98.0 | 99.8 | 101.1 |
| **pCTCL** | 88.4 | 91.2 | 92.4 | 92.1 | 92.3 | 96.2 |
| **PNK/TCL** | 52.0 | 71.5 | 79.1 | 81.3 | 80.7 | 83.3 |
| **ALL/LL** | 68.1 | 82.1 | 88.9 | 92.0 | 93.3 | 95.0 |
| **AML** | 24.6 | 52.9 | 69.3 | 78.7 | 84.8 | 89.8 |
| **CML** | 87.5 | 89.6 | 89.8 | 91.1 | 92.9 | 93.1 |
| **PV** | 95.5 | 95.0 | 94.5 | 92.9 | 89.6 | 89.5 |
| **ET** | 93.5 | 92.7 | 90.6 | 88.5 | 89.6 | 89.8 |
| **PMF** | 52.3 | 52.0 | 58.0 | 59.6 | 63.9 | 63.8 |
| **Other MPN** | 74.3 | 79.4 | 83.8 | 88.4 | 94.9 | 98.5 |
| **MCN** | 92.7 | 95.5 | 92.6 | 92.1 | 95.2 | 95.8 |
| **MDS** | 46.9 | 53.3 | 58.9 | 63.4 | 66.1 | 67.0 |
| **MDS/MPN** | 45.1 | 49.1 | 54.0 | 59.4 | 67.2 | 67.1 |
| **HDCN** | 87.6 | 91.5 | 92.8 | 93.6 | 95.6 | 97.3 |
| **HM** | **68.8** | **79.3** | **82.9** | **84.9** | **86.1** | **87.0** |

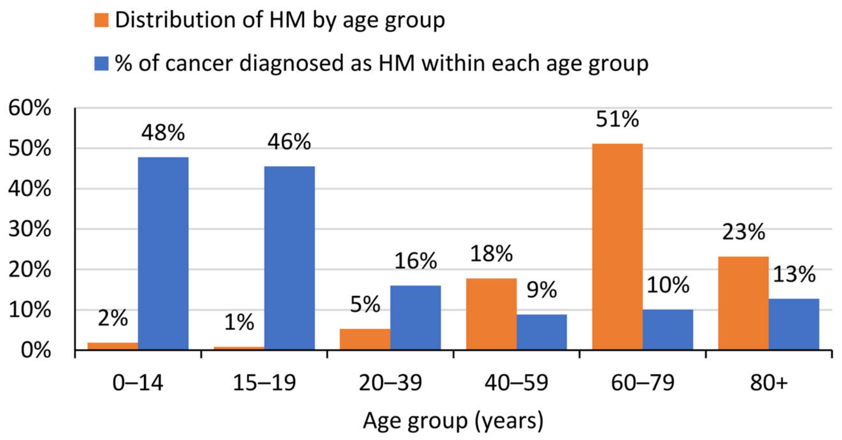


**Figure S1**: Proportion of HM diagnoses (N) by age group, Belgium 2018
